# Effects of Chronic Exposure to Sodium Arsenite on Expressions of VEGF and VEGFR2 Proteins in the Epididymis of Rats

**DOI:** 10.1155/2017/2597256

**Published:** 2017-07-16

**Authors:** Dai Yan-Ping, Gao Xiao-Qin, Ma Xiao Ping, Yue Ying Quan

**Affiliations:** ^1^Department of Histology and Embryology, Guizhou Medical University, Guiyang, Guizhou 550004, China; ^2^People's Hospital in Yueyanglou District, Yueyang, Hunan 414000, China; ^3^Department of Histology and Embryology, Zunyi Medical College, Zunyi, Guizhou 563000, China

## Abstract

**Objective:**

To study the expressions of VEGF and VEGFR2 at protein level in the epididymis of rats with arsenism.

**Methods:**

Forty male Sprague-Dawley rats were randomly divided into four groups: the high dose arsenic infected group (60.0 mg/L in water), the middle dose arsenic infected group (12.0 mg/L in water), the low dose arsenic infected group (2.4 mg/L in water), and the control group (distilled water). Rats were treated with arsenic through drinking water for 6 consecutive months. At the end of the experiment, the average densitometry values of apoptotic cells in epididymis tubules were determined by TUNEL method; the protein and mRNA levels of VEGF and VEGFR2 were observed by immunohistochemistry, Western blot, and real time fluorescent quantitative PCR, respectively.

**Results:**

Compared with the control group, in each infected group, the average densitometry values of apoptotic cells in the epididymis tubules were significantly lower. Compared with control group, protein and mRNA levels of VEGF and VEGFR2 in each infected group were obviously declined. The correlations between protein and mRNA levels of VEGF and VEGFR2 were positively exhibited (*r* = 0.843, 0.869,* p* < 0.05).

**Conclusions:**

Arsenism affects the expressions of VEGF and VEGFR2 in the epididymis of rats and results in apoptosis of pathophysiology of male infertility.

## 1. Introduction

Arsenic, a widely distributed element in nature, is an environmental toxin and human carcinogen [1]. A large number of studies have proved that arsenic is a multisite carcinogen in human, causing tumors in a variety of tissues including liver and lung [2–4]. The latest research suggests arsenic is a kind of environmental estrogen (EE), which affects the endocrine system, nervous system, reproduction, and development in the body. A study has found the interference effects of EEs on reproductive function, in which environmental endocrine disruptors have significant effects on estrogen and testosterone, leading to metabolic disorders and reproductive dysfunction [5]. However, arsenic as a carcinogen remains an enigma. On the one hand, it is definitively active in humans, whereas on the other hand, carcinogenesis in rodent models has never been convincingly demonstrated. The actual molecular events resulting in male reproductive toxicity from exposure of inorganic arsenic remain unclear [6]. Epididymis is a small organ located downstream of the testis. Spermatozoa is produced by the testis and acquired their capacity in the epididymis [7–11]. Epididymal sperm maturation is, therefore, essential for the establishment of male fertility. Vascular endothelial growth factor (VEGF) and VEGF receptor 2 (VEGFR2) were involved in the occurrence of the mature sperm. In this study we established the rat model with chronic arsenic poisoning and investigated expression levels of VEGF and VEGFR2 and apoptotic rates in epididymis of rats, in order to provide scientific evidence for the pathophysiology of the epididymis of rats influenced by chronic arsenic.

## 2. Materials and Methods

### 2.1. Experimental Animals

Forty, healthy clean level SD (Sprague–Dawley) male rats, weighing 160~200 gm were provided by the Guizhou Medical University Laboratory Animal Center, animal license: SCK (Guizhou), 2002-0001. The animals were housed singly per cage under controlled condition of ambient temperature (20~25°C), humidity (60~67%), and photoperiod controlled room (light : dark: 12 h : 12 h). After adaptive feed for one week, according to previous findings in this research, all the animals were divided equally into 4 groups, respectively, for the high (60.0 mg/L in water), middle (12.0 mg/L in water), and low (2.4 mg/L in water) dose arsenic infected group and the control group (distilled water), with 10 animals per group and their initial body weight were recorded along with a record of their daily water consumption. During the experiment, rats can freely feed and drink water. All the treatments were continued for six months.

### 2.2. The Main Reagent and Instrument

Sodium arsenite was obtained from Beijing Chemical Co. (pure analysis, Beijing Chemical Factory), cell apoptosis detection kit from Wuhan Boster Biotechnology Company, VEGF and VEGFR2 rabbit anti-rat polyclonal antibody from Wuhan Boster Biotechnology Company, SynGene Genius UV gel imaging system from Bio-Rad Company, USA, and Image J analysis software from Bio-Rad company, USA.

### 2.3. The Observation of Cell Apoptosis in Epididymis of Rats

For the detection of apoptosis, paraffin-embedded sections were stained with the TUNEL technique using an in situ apoptosis detection kit according to the instructions. To assess apoptosis in epididymis, 200 different epididymis tubules were observed in predetermined different fields in each section at magnification of ×400; the average densitometry values of apoptotic cell nucleus were determined by MIAS image analysis software.

### 2.4. Immunohistochemical Staining Method

For immunohistochemistry, 5 *μ*m of sections was mounted onto coated slides, dewaxed, and rehydrated. Antigen retrieval was performed by pressure cooking slides for 15 min in 0.01 M citrate buffer (PH 6.0); slides were incubated for 5 min in 3% hydrogen peroxide in methanol to block endogenous peroxidase activity and then washed in Tris-buffered saline (TBS). Nonspecific binding sites were blocked with an appropriate normal serum diluted 1 : 5 in TBS containing 5% bovine serum albumin before the addition of rabbit polyclonal antibody (VEGF, VEGFR2) and overnight incubation at 4°C. After washing in TBS, slides were incubated with the appropriate secondary antibody conjugated to biotin goat anti-rabbit for 30 min, at 37°C diluted 1 : 500 in the blocking mixture. Immunostaining was developed by application of diaminobenzidine (DAB) for 3–5 min, and slides were counterstained with hematoxylin, dehydrated, and mounted using Pertex mounting medium. Tan particles appearing in the cytoplasm or nucleus were positive cells. 10 different slices were randomly selected from each group and were observed in predetermined 5 different fields in each section at a magnification of ×400; the average densitometry values of positive cells were determined by Image J analysis software.

### 2.5. Protein Immunoblot (Western Blot) Method to Detect the Expression of VEGF and VEGFR2 Protein Levels

Western blotting was performed using epididymis lysates. In brief, protein extracts from each sample were added to a gel loading buffer and boiled for 10 min. Proteins (20 *μ*g/sample) in loading buffer were subjected to electrophoresis in 12% SDS-polyacrylamide gel for 2.5 h. The gel was transferred electrophoretically onto a polyvinylidene fluoride membrane and blocked in 5% nonfat powdered milk in Dulbecco's PBS for 2 h. The membranes were incubated overnight at 4°C with following primary antibodies: VEGF, VEGFR2, and GAPDH were used as a loading control for total protein. After being washed with TBST (Tris buffered saline) with Tween three times for 10 min each, the membranes were incubated with goat anti-rabbit IgG antibody for 2 h. The membranes were then washed for three times for 10 min each, followed by signal development by using an enhanced chemiluminescence (ECL) detection kit from Pierce. Image J software (nih) was used to scan and analyze densitometry values.

### 2.6. Real Time Fluorescent Quantitative PCR

One microgram of total RNA from each sample was reverse transcribed into first-strand cDNA with random hexamers using SuperScript III Reverse Transcriptase. Primer sets for all genes were purchased from Wuhan Boster Biotechnology Company, VEGF: F: 5′-GCCCACTGAGGAGTCCAACA-3′; R: 5′-TCCTATGTGCTGGCCTTGGT-3′; VEGFR2: F: 5′-GGCACCACTCAAACGCTGAC-3′; R: 5′-CCTCTCTCCTCTCCCGACTT-3′; *β*-actin: F: 5′-TCATGTTTGAGACCTTCAA-3′; R: 5′-GTCTTTGCGGATGTCCACG-3′; PCR was performed using SYBR Premix Ex Taq with the ABI PRISM 7900HT according to the instructions of manufacturer. Experimental samples were matched to a standard curve generated by amplifying serially diluted products using the same PCR protocol. To correct for variability in RNA recovery and efficiency of reverse transcription, *β*-actin cDNA was amplified and quantified in each cDNA.

The reaction system was as follows: the iQ™SYBR was 12.5 *μ*L, the forward primer was 1.0 *μ*L, the reverse primer was 1.0 *μ*L, the ddH_2_O_2_ was 8 *μ*L, the DNA template was 2.5 *μ*L, and the total volume was 25 *μ*L. Reaction conditions were as follows: 95°C predegenerated for 10 min, 95°C degenerated for 10 s, 60°C annealed for 30 s, and 72°C extended for 34 s, circulated for forty times. *β*-actin was internal, Ct value of detecting various template (C represented the cycle; T represented threshold). The smaller the Ct, the higher the expression.

### 2.7. Statistical Analysis

Statistical analysis was performed with SPSS 13.0 software. Date were presented as mean +/− standard deviation (X +/− S) for description. One-way ANOVA was used for multiple sets of average comparison, Bonferroni test was used for average comparison between the two groups, significant level is *α* = 0.05, and *p* < 0.05 was considered statistically significant. The data shown in the figures were representative of 3 or 5 independent experiments.

## 3. Results

### 3.1. The Expression of VEGF and VEGFR2 in the Epididymis of Rats

**Figure 1:**
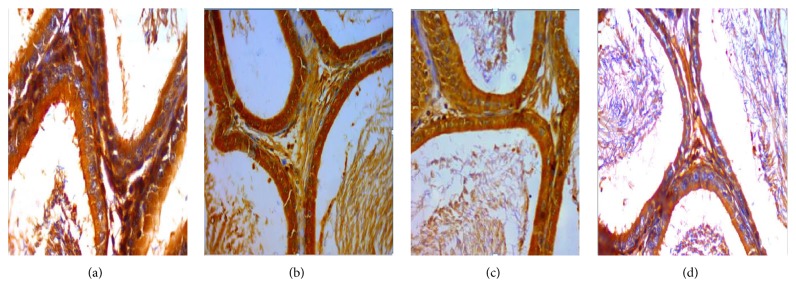
(a), (b), (c), (d) Immunohistochemical expression of VEGF in epididymis corpus of rats (SP ×400). (a) Control group, (b) low dose group, (c) middle dose group, and (d) high dose group.

**Figure 2:**
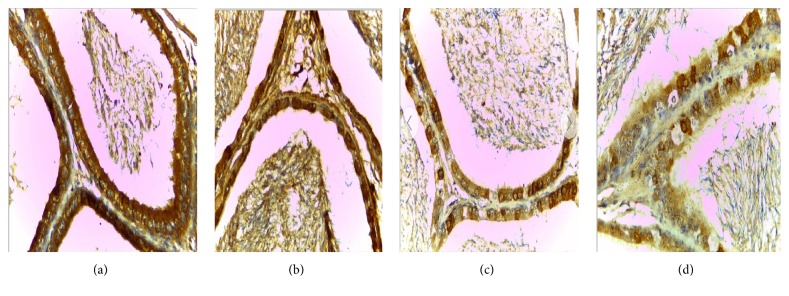
(a), (b), (c), (d) Immunohistochemical expression of VEGF in epididymis caput of rats (SP ×400). (a) Control group, (b) low dose group, (c) middle dose group, and (d) high dose group.

**Figure 3:**
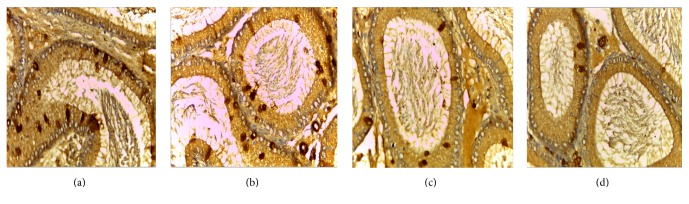
Immunohistochemical expression of VEGF in epididymis cauda of rats (SP ×400). (a) Control group, (b) low dose group, (c) middle dose group, and (d) high dose group.

VEGF is expressed in all master cell's cytoplasm of epididymis pipe. VEGF was expressed the strongest in corpus of epididymis, weaker at the caput, and weakest at the cauda (Figures 1, 2, and 3).

**Figure 4:**
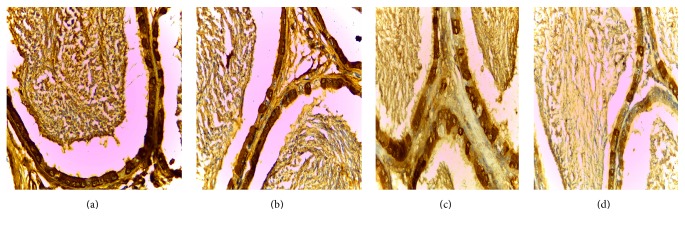
(a), (b), (c), (d) Immunohistochemical expression of VEGFR2 in epididymis caput of rats (SP ×400). (a) Control group, (b) low dose group, (c) middle dose group, and (d) high dose group.

**Figure 5:**
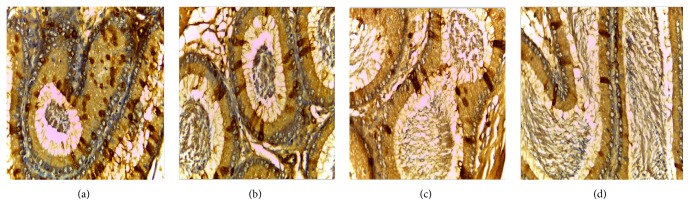
(a), (b), (c), (d) Immunohistochemical expression of VEGFR2 in epididymis cauda of rats (SP ×400). (a) Control group, (b) low dose group, (c) middle dose group, and (d) high dose group.

VEGFR2 is expressed in all master cell's cytoplasm of epididymis pipe. VEGFR2 was expressed the strongest at the cauda and the caput followed, but positive expression was not seen in the corpus (Figures 4 and 5).

Positive cells of VEGF and VEGFR2 mainly located in cytoplasm of epididymal epithelial cells, part of the interstitial small vascular endothelial cell, and blood capillary endothelial cell were positive (Figures 1, 2, 3, 4, and 5).

Results of immunohistochemistry showed that, compared with the control group, the protein levels of VEGF and VEGFR2 in infected group were lower and the differences were statistically significant (*p* < 0.05). Comparing between the groups, the differences in the protein levels of VEGF in epididymis corpus, caput, and cauda of rats were statistically significant (*F* = 64.750, 29.500, and 22.150, all *p* < 0.05) (Tables 1 and 2). Comparing between the groups, the differences in protein levels of VEGFR2 protein in epididymis caput and cauda of rats were statistically significant (*F* = 41.000, 213.00, all *p* < 0.05) (Tables 1 and 2).

### 3.2. Apoptosis Cells of Rat Epididymis Tissue

**Figure 6:**
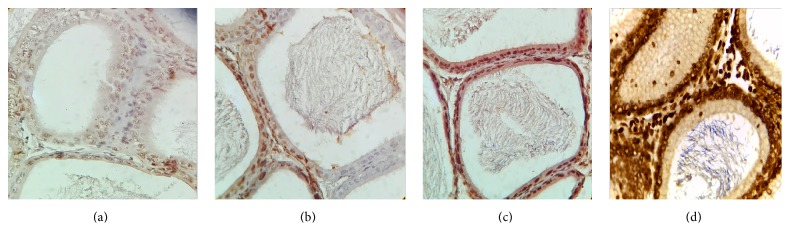
Nucleus apoptosis in epididymis caput of rats (TUNEL staining ×400).* Note*. (a) Control group, (b) low dose group, (c) middle dose group, and (d) high dose group.

**Figure 7:**
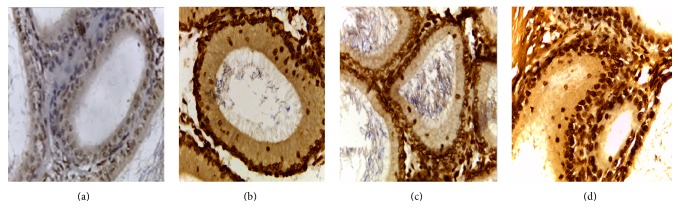
(a), (b), (c), (d) Nucleus apoptosis in epididymis corpus of rats (TUNEL staining ×400).* Note*. (a) Control group, (b) low dose group, (c) middle dose group, and (d) high dose group.

**Figure 8:**
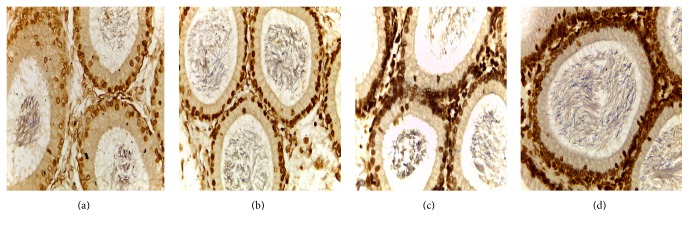
(a), (b), (c), (d) Nucleus apoptosis in epididymis cauda of rats (TUNEL staining ×400).* Note*. (a) Control group, (b) low dose group, (c) middle dose group, and (d) high dose group.

 The changes of arsenic poisoning on the apoptotic cell nucleus in epididymal tubes of rats (Table 3): results of TUNEL staining showed that a small amount of apoptotic cells in epididymis was seen in control group (Figures 6, 7, and 8). Epithelial apoptotic cells in low, middle, and high dose group were increased gradually, the nuclei were hyperchromatic, the volume was much smaller than normal cells, condensed chromatin gathered under nuclear membrane, and tan particles were presented. The expression at the corpus was the strongest, at the caput weaker, and at the cauda the weakest.

Compared with the control group, in the high, middle, and low doses arsenic infected groups, the average densitometry values of apoptotic cells in the epididymis tubules were significantly lower. With the increase of arsenic infected doses, the average densitometry showed a decreasing trend (Table 3). Compared between the groups, the differences in average densitometry values of apoptotic cells in epididymis caput, corpus, and cauda of rats were statistically significant (*F* = 48.667, 17.000, and 13.000, all *p* < 0.05).

### 3.3. The Effects of Arsenic Poisoning on Protein Levels of VEGF and VEGFR2 in Epididymis of Rats (Figures 9, 10, and 11 and Table 4)

Compared with the control group, the protein levels of VEGF and VEGFR2 in epididymis of rats were lower; differences were statistically significant (*p* < 0.05). Compared between the groups, the protein levels of VEGF and VEGFR2 in epididymis of rats were lower; the differences were statistically significant (*F* = 28.668, 31.213, all *p* < 0.05) (Table 4). The correlations between protein levels of VEGF and VEGFR2 were positively exhibited (*r* = 0.843, *p* < 0.05).

### 3.4. The mRNA Levels of VEGF and VEGFR2 in Epididymis of Rats

Compared with the control group, the mRNA levels of VEGF and VEGFR2 in each infected group were lower; the differences were statistically significant (*F* = 7.888, 7.088, all *p* < 0.05) (Table 5). The correlations between mRNA levels of VEGF and VEGFR2 were positively exhibited (*r* = 0.869, *p* < 0.05).

## 4. Discussion

It is known that inorganic arsenic exists in both a pentavalent form arsenite and a trivalent form arsenite, the latter being more toxic; both valence forms of arsenic undergo enzymatic methylation [12, 13]. Arsenic, as an environmental agent, is considered to be a very high priority toxic substance largely due to its carcinogenic potential in humans [14, 15]. Humans are exposed to arsenic mainly through oral or inhalation routes. Oral exposure occurs via consumption of contaminated water, food, and so on. Occupational exposure, on the other hand, occurs mainly by burning of arsenic contaminated coal [16]. Chronic exposure may exert reproductive system disorders and cancer [17]. Studies have proved that arsenic has endocrine disruption [18].

Epididymis is a long and single tubule that is organized into four major segments, respectively, for the initial segment, the caput, the corpus, and the cauda. Epididymides are further divided into intrasegmental regions that are delineated by connective tissue septa and express a distinct set of genes. Epithelial cells with specific functions and morphological characteristics are located in these regions; they form the blood-epididymis barrier [19, 20] and establish a unique luminal microenvironment for the concentration, maturation, and storage of spermatozoa. Approximately 10% to 15% of infertile men suffer from azoospermia [10, 21, 22]. An elaborate communication network between these cells contributes to the regulation of various transport mechanisms in the epididymis [11, 23–26].

VEGF is one of the important growth factors related to reproductive system. VEGF can promote angiogenesis. Scholars reported VEGF may also play an important role in resistance to apoptosis. The receptors of VEGF include VEGFR-1, VEGFR-2, and VEGFR-3; after VEGF combined with VEGFR2, it can promote cell division and antiapoptotic [27]. VEGF and VEGFR2 are involved in the occurrence of the mature sperm, and VEGF is a soluble promoting angiogenesis factor and the specificity mitosis factor of the endothelial cells, which can induce the division and migration of endothelial cell and are expressed in normal tissue. It has been found that VEGF and VEGFR2 are the main regulators of angiogenesis. Studies have showed that high expression of VEGF in testis and epididymis can lead to angiogenesis and may be associated with male infertility. Scholars have found that VEGF has the function of inhibiting cell apoptosis, which indicate VEGF may act as a protective factor involved in the mechanism of chronic arsenic poisoning. Some scholars [28] reported VEGF and VEGFR2 were expressed in the testis, epididymis, seminal vesicle, and prostate gland tissues, which may be related to male reproductive system. However, whether VEGF and VEGFR2 through the effect on cardiovascular system of testis and epididymis or direct effects on reproductive cells affect the incidence of mature sperm is still not clear. Mary has found that varicocele has influences on the expression of VEGF and VEGFR2 in epididymis of rats, indicating it may have a certain relationship with male infertility [29].

Currently, research on male infertility caused by arsenic poisoning is more concentrated on the testicle, while epididymis tissue coverage is less. Researchers found that epididymis plays an important role in movement, improvement, and maturity of sperm [30]. Epididymis dysfunction is an important cause of male infertility. Study of VEGF and VEGFR2 in arsenic poisoning has not yet been reported.

In this study, the immunohistochemical results showed that VEGF was expressed the strongest in corpus of epididymis, the caput followed, and it was expressed the weakest at the cauda. VEGFR2 was expressed the strongest at the cauda, the caput followed, and positive expression was not seen in the corpus. VEGF and VEGFR2 positive staining mainly located in the epididymis epithelial cell cytoplasm, part of the interstitial small vascular endothelial cell, and blood capillary endothelial cell also showed masculine gender, consistent with Mary and other findings [31]. Compared with the control group, in the high, middle, and low doses arsenic infected groups, the average densitometry values of apoptotic cells in the epididymis tubules were significantly lower. With the increase of arsenic infected doses, the average densitometry showed a decreasing trend. Comparing between the groups, the differences in the average densitometry values of apoptotic cells in the caput, corpus, and cauda of epididymis were statistically significant (*p* < 0.05).

Apoptosis is a necessary process for maintaining normal physiological function of human. In pathological conditions, it can lead to disease, tumor cell apoptosis. Our previous studies have found that arsenic poisoning can cause sperm apoptosis [32]. According to the results of this study compared with control group, the average densitometry values of apoptotic nuclei in epididymal tubes of each infected group were lower, which clearly indicate that arsenic poisoning can cause cell apoptosis in epididymis, and with the increase of infected dose, epididymal epithelial cells apoptosis level was on the rise. Compared with control group, protein and mRNA levels of VEGF and VEGFR2 in each infected group were obviously declined. The correlations between protein and mRNA levels of VEGF and VEGFR2 were positively exhibited (*r* = 0.843, 0.869, *p* < 0.05), it may be associated with the dose of arsenic, and the expression of VEGFR2 was induced by VEGF.

From the present study, it may be suggested that sodium arsenite treatment impaired male reproductive systems. In a word, we provide strong evidence that expressions of VEGF and VEGFR2 may affect the microenvironment of epididymis and the maturation of sperm. Our study suggests that it might be one of the mechanisms of male infertility caused by arsenic poisoning. However, the exact mechanism deserves additional study.

At present, researches on the expression and function about VEGF and VEGFR2 in the reproductive system have made great progress, and it has been fully indicated that VEGF and blood vessel formation were playing an important role in the process of male reproductive system. These studies may provide us with insight into the molecular mechanisms of arsenic's transport and toxicity, as well as the complete understanding of arsenic carcinogenesis. Promoting the antiapoptotic effect of VEGF may play a very important role in effective control and prevention of arsenic reproductive toxicity. VEGF and VEGFR2 may be target of male pill that will bring new hope for the development of male contraceptives. In a word, the stories about VEGF and male infertility caused by arsenic poisoning will continue.

## Figures and Tables

**Figure 9:**
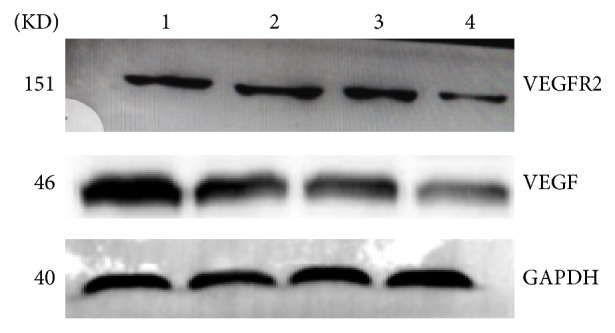
The electrophoresis figure of protein levels of VEGF and VEGFR2 in arsenic poisoning epididymis of rats. The protein expression levels of VEGF and VEGFR2 were analyzed by western blot, Figure 7, Lanes 1–4, 0 mg/L, 2.4 mg/L, 12 mg/L, and 60 mg/L arsenic infected group.

**Figure 10:**
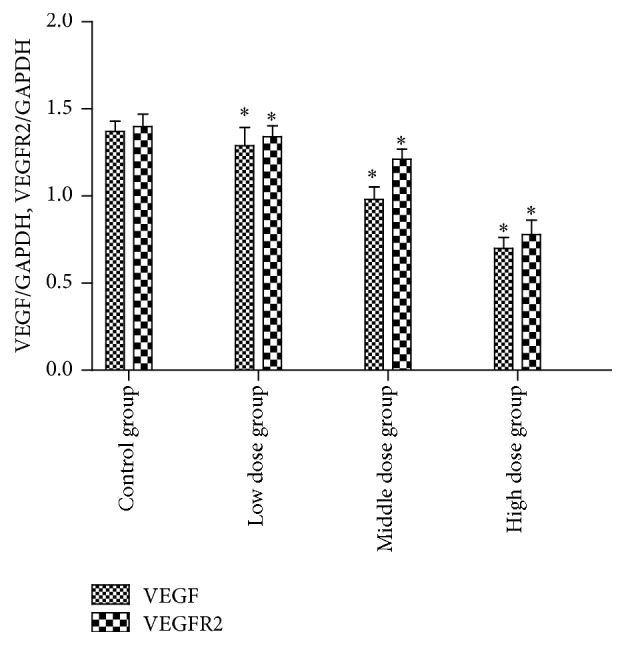
VEGF and VEGFR2 protein expression investigated by western blotting. The average densitometry values measurement of VEGF and VEGFR2 to GAPDH ratios, *n* = 3, x ± s, *∗* compared with the control group, and *p* < 0.05.

**Figure 11:**
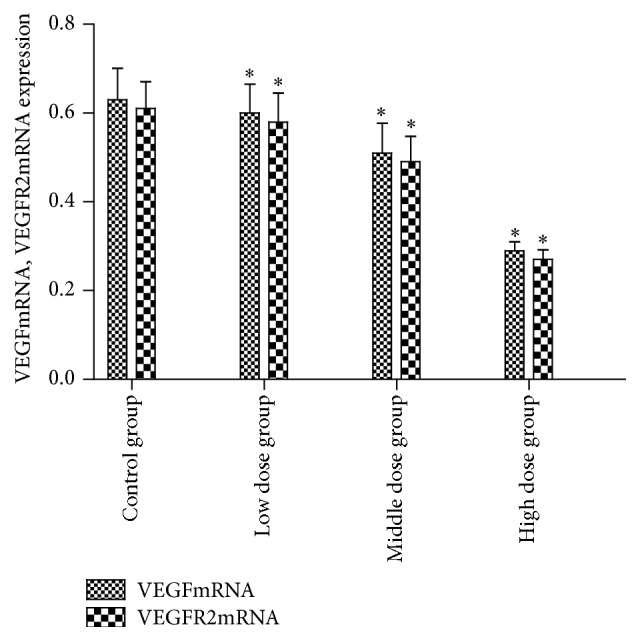
The effects of arsenic poisoning on protein levels of VEGF and VEGFR2 in epididymis of rats (Figures 9 and 10 and Table 4). The mRNA levels of VEGF and VEGFR2 to *β*-actin ratios, *n* = 3, x ± s, *∗* compared with the control group, and *p* < 0.05.

**Table 1:** Immunohistochemistry image analysis results of VEGF protein in arsenic infected epididymis. (*n* = 10, x ± s).

Group	The corpus	The caput	The cauda
Control group	0.170 ± 0.003	0.240 ± 0.003	0.284 ± 0.025
Low dose group	0.132 ± 0.002^*∗*^	0.210 ± 0.002^*∗*^	0.260 ± 0.050^*∗*^
Middle dose group	0.103 ± 0.008^*∗*^	0.190 ± 0.003^*∗*^	0.234 ± 0.035^*∗*^
High dose group	0.087 ± 0.004^*∗*^	0.130 ± 0.001^*∗*^	0.223 ± 0.170^*∗*^

*Note*. *∗* compared with the control group, *p* < 0.05.

**Table 2:** Immunohistochemistry image analysis results of VEGFR2 protein in arsenic infected epididymis (*n* = 10, x ± s).

Group	The caput	The cauda
Control group	0.204 ± 0.007	0.241 ± 0.019
Low dose group	0.173 ± 0.003^*∗*^	0.076 ± 0.003^*∗*^
Middle dose group	0.124 ± 0.007^*∗*^	0.060 ± 0.008^*∗*^
High dose group	0.121 ± 0.019^*∗*^	0.052 ± 0.006^*∗*^

*Note*. *∗* compared with the control group, *p* < 0.05.

**Table 3:** Effect of arsenic on nucleus apoptosis in epididymis of rats (*n* = 10, x ± s).

Group	The corpus	The caput	The cauda
Control group	0.179 ± 0.015	0.145 ± 0.005	0.114 ± 0.055
Low dose group	0.185 ± 0.001^*∗*^	0.163 ± 0.006^*∗*^	0.129 ± 0.002^*∗*^
Middle dose group	0.201 ± 0.011^*∗*^	0.195 ± 0.014^*∗*^	0.144 ± 0.003^*∗*^
High dose group	0.287 ± 0.018^*∗*^	0.197 ± 0.059^*∗*^	0.163 ± 0.135^*∗*^

*Note*. *∗* compared with the control group, *p* < 0.05.

**Table 4:** Effects of arsenic poisoning on protein levels of VEGF and VEGFR2 in epididymis of rats (*n* = 3, x ± s).

Group	VEGF/GAPDH	VEGFR2/GAPDH
Control group	1.370 ± 0.060	1.400 ± 0.070
Low dose group	1.290 ± 0.104^*∗*^	1.340 ± 0.063^*∗*^
Middle dose group	0.980 ± 0.072^*∗*^	1.210 ± 0.060^*∗*^
High dose group	0.700 ± 0.063^*∗*^	0.780 ± 0.082^*∗*^

*Note*. *∗* compared with the control group, *p* < 0.05.

**Table 5:** Effects of arsenic poisoning on mRNA levels of VEGF and VEGFR2 in epididymis of rats (*n* = 3, x ± s).

Group	VEGF/*β*-actin	VEGFR2/*β*-actin
Control group	0.63 ± 0.071	0.61 ± 0.060
Low dose group	0.60 ± 0.065^*∗*^	0.58 ± 0.065^*∗*^
Middle dose group	0.51 ± 0.067^*∗*^	0.49 ± 0.058^*∗*^
High dose group	0.29 ± 0.020^*∗*^	0.27 ± 0.022^*∗*^

*Note*. *∗* compared with the control group, *p* < 0.05.
