# Corrigendum to “Effects of Chronic Exposure to Sodium Arsenite on Expressions of VEGF and VEGFR2 Proteins in the Epididymis of Rats”

**DOI:** 10.1155/2017/4284156

**Published:** 2017-10-25

**Authors:** Dai Yan-Ping, Gao Xiao-Qin, Ma Xiao Ping, Yue Ying Quan

**Affiliations:** ^1^Department of Histology and Embryology, Guizhou Medical University, Guiyang, Guizhou 550004, China; ^2^People's Hospital in Yueyanglou District, Yueyang, Hunan 414000, China; ^3^Department of Histology and Embryology, Zunyi Medical College, Zunyi, Guizhou 563000, China

In the article titled “Effects of Chronic Exposure to Sodium Arsenite on Expressions of VEGF and VEGFR2 Proteins in the Epididymis of Rats” [1], there was an error in the Acknowledgments section, which should be corrected as follows:

This study was supported by Guizhou Province Science and Technology Fund (Qian talents team [2014] no. 4005); Benefits of Science and Technology Plan (according to city fellow word [2014] no. 01); Guizhou Province Science and Technology Fund (Qian LH word) [2015] no. 7568.

## References

[1] Yan-Ping D., Xiao-Qin G., Ping M. X., Quan Y. Y. (2017). Effects of Chronic Exposure to Sodium Arsenite on Expressions of VEGF and VEGFR2 Proteins in the Epididymis of Rats. *BioMed Research International*.

