# Corrigendum #2 to “Effects of Chronic Exposure to Sodium Arsenite on Expressions of VEGF and VEGFR2 Proteins in the Epididymis of Rats”

**DOI:** 10.1155/2018/1082961

**Published:** 2018-11-28

**Authors:** Dai Yan-Ping, Gao Xiao-Qin, Ma Xiao-Ping, Yue Ying-Quan

**Affiliations:** ^1^Department of Histology and Embryology, Guizhou Medical University, Guiyang, Guizhou 550004, China; ^2^People's Hospital in Yueyanglou District, Yueyang, Hunan 414000, China; ^3^Department of Histology and Embryology, Zunyi Medical and Pharmaceutical College, Zunyi, Guizhou 563000, China

In the article titled “Effects of Chronic Exposure to Sodium Arsenite on Expressions of VEGF and VEGFR2 Proteins in the Epididymis of Rats” [1], there was an error in the third affiliation. The corrected authors' list and affiliations are shown above.

## References

[1] Yan-Ping D., Xiao-Qin G., Ping M. X., Quan Y. Y. (2017). Effects of chronic exposure to sodium arsenite on expressions of VEGF and VEGFR2 proteins in the epididymis of rats. *BioMed Research International*.

